# Role of tumor necrosis factor-α in the mortality of hospitalized patients with severe and critical COVID-19 pneumonia

**DOI:** 10.18632/aging.203663

**Published:** 2021-11-01

**Authors:** Fang Jia, Gang Wang, Jing Xu, Junhong Long, Fuxue Deng, Wei Jiang

**Affiliations:** 1 Department of Endocrinology, The Second Affiliated Hospital of Xi’an Jiaotong University, Xi’an, Shaanxi, P.R. China; 2 Department of Critical Care Medicine, The Second Affiliated Hospital of Xi’an Jiaotong University, Xi’an, Shaanxi, P.R. China; 3 Department of Cardiology, The Second Affiliated Hospital of Xi’an Jiaotong University, Xi’an, Shaanxi, P.R. China

**Keywords:** COVID-19, TNF-alpha, mortality, cytokine storm, illness severity

## Abstract

The coronavirus disease 2019 (COVID-19) is presently the most pressing public health concern worldwide. Cytokine storm is an important factor leading to death of patients with COVID-19. This study aims to characterize serum cytokines of patients with severe or critical COVID-19. Clinical records were obtained from 149 patients who were tested at the Sino-French New City Branch of Tongji Hospital from 30 January to 30 March 2020. Data regarding the clinical features of the patients was collected and analyzed. Among the 149, 45 (30.2%) of them had severe conditions and 104 (69.8%) of that presented critical symptoms. In the meantime, 80 (53.7%) of that 149 died during hospitalization. Of all, male patients accounted for 94 (69.1%). Compared with patients in severe COVID-19, those who in critical COVID-19 had significantly higher levels of tumor necrosis factor-α (TNF-α), interleukin-6 (IL-6), IL-8, and IL-10. Moreover, the passed-away patients had considerably higher levels of TNF-α, IL-6, IL-8, and IL-10 than those survived from it. Regression analysis revealed that serum TNF-α level was an independent risk factor for the death of patient with severe conditions. Among the proinflammatory cytokines (IL-1β, TNF-α, IL-8, and IL-6) analyzed herein, TNF-α was seen as a risk factor for the death of patients with severe or critical COVID-19. This study suggests that anti-TNF-α treatment allows patients with severe or critical COVID-19 pneumonia to recover.

## INTRODUCTION

As coronavirus disease 2019 (COVID-19) spreads, the World Health Organization (WHO) declared being pandemic disease on March 11, 2020. COVID-19 is a clinical syndrome caused by the severe acute respiratory syndrome coronavirus 2 (SARS-Cov-2), which is one of the seven identified human coronaviruses [1]. The SARS-Cov-2 and the other two epidemic coronaviruses including severe acute respiratory syndrome (SARS) and Middle East respiratory syndrome are sharing over 50% of genome sequences [2]. As of July 08, 2021, the WHO has reported that 184,572,371 persons have been infected and 3,997,640 patients have died worldwide owing to the highly contagious nature of SARS-Cov-2. Nevertheless, most of the patients undergo mild to moderate performance just like common cold, but a high mortality was found in some patients with severe or critical conditions, especially in older patients with previous chronic diseases [3]. The epidemiological and clinical characteristics of COVID-19 must be elucidated to improve the diagnosis and therapy and reduce the mortality of this highly infectious disease.

The angiotensin-converting enzyme 2 (ACE 2) is the SARS-Cov-2 receptor on cell surface. Virus enters and replicates in the infected cell cytoplasm through binding to ACE 2, and then the cell disintegrates and releases further virions to attack other cells [4]. In general, upon activating the viral antigen signal from antigen presentation cells, the immune cells rapidly synthesize and release plenty of proinflammatory cytokines to develop immune defense. However, in some conditions, excess cytokines trigger cytokine storm, and therefore, develop an overactive immune response, leading to systemic inflammatory response, multiple organ dysfunction and death [5, 6].

The pathological findings showed that plenty of monocytes and macrophages cells being secreted and diffused in lungs. In some patients with severe COVID-19 pneumonia, the described above may be the reason for increasing proinflammatory cytokines, such as interleukin (IL)-6, IL-1β, IL-8, and tumor necrosis factor (TNF)-α, signaling the initial phase of cytokine storm [7]. The pathologic process begins with the recognition of viral antigens, followed by the increase in macrophage cells in target organs and the activation of the immune system, resulting in cytokine storm, and then death [8]. In the present study, the clinical features and serum cytokines of patients with severe or critical COVID-19 pneumonia were analyzed to find more efficient ways for these patients.

## MATERIALS AND METHODS

### Participants and definitions

The clinical data of 149 patients with severe or critical COVID-19 pneumonia hospitalized in the Sino-French New City Branch of Tongji Hospital from 30 January to 30 March 2020 were analyzed. The patients were diagnosed with SARS-CoV-2 infection by twice COVID-19 nucleic acid test positive. According to the guideline for diagnosis and treatment of COVID-19 infection, the participants were classified into two groups [9]. Patients with any of the following conditions were assigned to severe group: oxygen saturation by pulse oximeter ≤ 93% in resting state, respiration rate ≥ 30 times/min, and arterial partial pressure of oxygen (PaO_2_)/ fraction of inspired oxygen ≤ 300 mmHg. Once patients develop symptom of shock, respiratory failure and requiring mechanical ventilation or with other organ failure during hospitalization, they were noticed with critical condition. The laboratory detections (e.g., blood biochemistry, immune parameters, and routine tests) were immediately performed upon admission. All patients’ conditions with the length of stay were documented.

This retrospective study was performed in accordance with the requirements of the Declaration of Helsinki. Given that patients were placed in quarantine as a precaution, informed consent was obtained by oral permission via telephone communication. All patient information had been de-identified when the data were collected and analyzed.

### Laboratory diagnosis and tests

All patients were collected upper respiratory tract throat swab or nasal swab specimens when they were newly admitted to our hospital, and immediately stored in the virus transport medium [10]. Sputum specimens were also collected when necessary. SARS-CoV-2 virus were detected using the TaqMan one step RT-PCR kits (Shanghai Huirui Biotechnology Co., Ltd, China and Shanghai BioGerm Medical Biotechnology Co., Ltd, China) following the supplier’s protocol. The plasma cytokines (TNF-α, IL-6, IL-8, IL-1β, and IL-10) were measured using the Human Th1/2 Cytokine Kit II (BD Biosciences, Franklin Lakes, NJ, USA) following the product protocol.

### Statistical analysis

Software SPSS version 18.0 was applied to perform the statistical analyses (IBM Corp., Armonk, NY, USA). Chi-square test was used for categorical variables comparisons. For continuous variables comparisons, Student’s *t*-test was used for normally distributed data, otherwise, Mann-Whitney U test was instead. Cox regression and logistic regression analysis were employed to examine the simultaneous effects of serum TNF-α level and several potential confounding factors on death and illness severity, respectively. Statistical significance was considered at *p* < 0.05.

### Ethical approval

All activities associated with this project were approved by the Ethics Committee of the Second Affiliated Hospital of Xi’an Jiaotong University.

### Data availability statement

The data that support the findings of this study are available from the corresponding authors, (F.D. and W.J.) upon reasonable request.

## RESULTS

### Clinical characteristics upon admission

149 severe or critical cases were included in this study. 45 (30.2%) with severe symptoms and 104 (69.8%) with critical COVID-19 pneumonia were characterized and presented in Table 1. The mean age was at the age of 64. Male patients accounted for 94 (69.1%). Fever (88.6%), cough (68.5%), and dyspnea (34.9%) were the most common symptoms. The elder (68.63±10.33 vs. 53.67±13.58, *p* < 0.001) and male (72/104 vs. 22/45, *p* = 0.018) patients presented critical conditions more frequently. For the patients with comorbidities (e.g., coronary artery disease [CAD], hypertension, chronic obstructive pulmonary disease [COPD], and diabetes mellitus [DM]), more patients with critical COVID-19 exhibited hypertension (47.6% vs. 24.4%, p = 0.008) and CAD (19.4% vs. 4.4%, *p*=0.019) than patients with severe one. COPD and DM showed no significant difference between severe and critical cases.

**Table 1:** Clinical characteristics of patients with severe or critical COVID-19.

**Characteristics**	**Total N=149**	**Severe N=45**	**Critical N=104**	***P* value**
Age (years)	64±13.29	53.67±13.58	68.63±10.33	<0.001
Sex(M/F)	94/55	22/23	72/32	0.018
DM (%)	16.8	15.6	17.5	0.774
Hypertension (%)	40.3	24.4	47.6	0.008
COPD (%)	6.7	2.2	8.7	0.146
CAD (%)	14.8	4.4	19.4	0.019
SaO_2_ (%)	87.77±12.64	94.62±4.20	84.81±13.88	<0.001
Fever (%)	88.6	88.9	88.5	0.940
Cough (%)	68.5	60.0	72.1	0.144
Chest distress/pain (%)	26.2	6.7	36	<0.001
Dyspnea (%)	34.9	15.6	43.3	0.001
Fatigue (%)	30.2	17.8	35.6	0.30
Dizzy/headache (%)	7.4	6.7	7.7	0.826
Nausea (%)	2.7	2.2	2.9	0.818
Breathe(times/min)	24.76±6.57	21.93±4.17	25.98±7.05	<0.001
Heart rate(times/min)	96.32±18.92	92.73±15.43	97.87±20.11	0.129
Systolic blood pressure (mmHg)	132.32±20.22	125.49±17.82	135.27±20.57	0.006
Diastolic blood pressure(mmHg)	79.97±13.23	80.27±14.74	79.84±12.60	0.856
WBC (×10^9^/L)	9.52±5.82	6.24±2.58	10.94±6.26	<0.001
Neutrophil (×10^9^/L)	8.12±5.52	4.68±2.60	9.61±5.80	<0.001
Lymphocyte (×10^9^/L)	0.77±0.47	1.07±0.47	0.64±0.41	<0.001
Hemoglobin (g/L)	125.16±21.48	123.49±18.95	125.86±22.53	0.534
Platelet (×10^9^/L)	195.10±96.76	239.18±105.4	176.03±86.56	<0.001
Blood urea nitrogen(mmol/L)	9.59±9.22	4.76±2.74	11.68±10.21	<0.001
Creatinine (μmol/L)	95.74±81.99	65.29±15.24	108.91±94.76	<0.001
eGFR (ml/min/1.73m^2^)	78.10±29.45	95.547±23.62	70.55±28.58	<0.001
Blood uric acid (μmol/L)	270.13±157.55	233.36±71.61	286.05±180.64	0.012
Alanine aminotransferase(U/L)	30(6-705)	23(6-221)	30(7-705)	0.165 ^a^
Aspartate aminotransferase (U/L)	38(10-2368)	27(12-189)	42(10-2368)	<0.001 ^a^
Albumin (g/L)	31.85±5.21	36.01±4.49	30.05±4.43	<0.001
ALP(IU/L)	85.49±41.26	77.04±39.76	89.14±41.56	0.100
TBil (μmol/L)	10.8(3.2-174.1)	9.2(3.3-49.0)	12(3.2-174.1)	0.001 ^a^
Total cholesterol (mmol/L)	3.57±0.99	3.83±0.89	3.47±1.01	0.041
LDH (IU/L)	488.36±311.44	285.53±94.67	576.12±331.35	<0.001
TNF-α(pg/ml)	9.2(4.0-69.7)	7.3(4.0-11.8)	10.3(4.0-69.7)	<0.001 ^a^
IL-6(pg/ml)	31.8(1.5-5000)	4.7(1.5-94.7)	48.4(2.8-5000)	<0.001 ^a^
IL-8(pg/ml)	20.6(5.0-1045)	9.3(5.0-86.1)	26.6(5.0-1045)	<0.001 ^a^
IL-1β(pg/ml)	5.0(5.0-82.0)	5.0(5.0-82.0)	5.0(5.0-14.8)	0.288 ^a^
IL-10(pg/ml)	5.8(5.0-73.7)	5.0(5.0-12.5)	10.2(5.0-73.7)	<0.001 ^a^
Death	80	0	80	<0.001

^a^: Used Mann-Whitney U test.

Abbreviations: SaO_2_, oxygen saturation; eGFR, estimated glomerular filtration rate; COPD, chronic obstructive pulmonary disease; CAD, coronary artery disease; DM, diabetes mellitus; WBC, white blood cells; ALP, alkaline phosphatase; TBil, total bilirubin; LDH, lactate dehydrogenase; CI, confidence interval.

### Laboratory findings upon admission

Upon admission, 90 (60.4%) cases presented oxygen saturation on oxygen inhalation less than (equally to) 93%, of whom 14 were in the severe group and 76 were in the critical group. In addition, oxygen saturation level significantly decreased in the critical patients (84.81±13.88 vs. 94.62±4.20, *p* < 0.001). As expected, the critical cases had a faster breathing rate than the severe ones (25.98±7.05 vs. 21.93±4.17, *p* < 0.001). Systolic blood pressure was significantly higher in the critical patients than in the severe patients (135.27±20.57 vs. 125.49±17.82, *p* = 0.006). The levels of white blood cells (WBC), neutrophils and most blood biochemical indicators (e.g., blood urea nitrogen, creatinine, blood uric acid, aspartate aminotransferase, lactate dehydrogenase [LDH], and total bilirubin [TBil]) were greatly higher in critical patients than those in severe cases. Moreover, the levels of lymphocytes, platelets, estimated glomerular filtration rate (eGFR), albumin and total cholesterol were lower in critical group.

**Figure 1:**
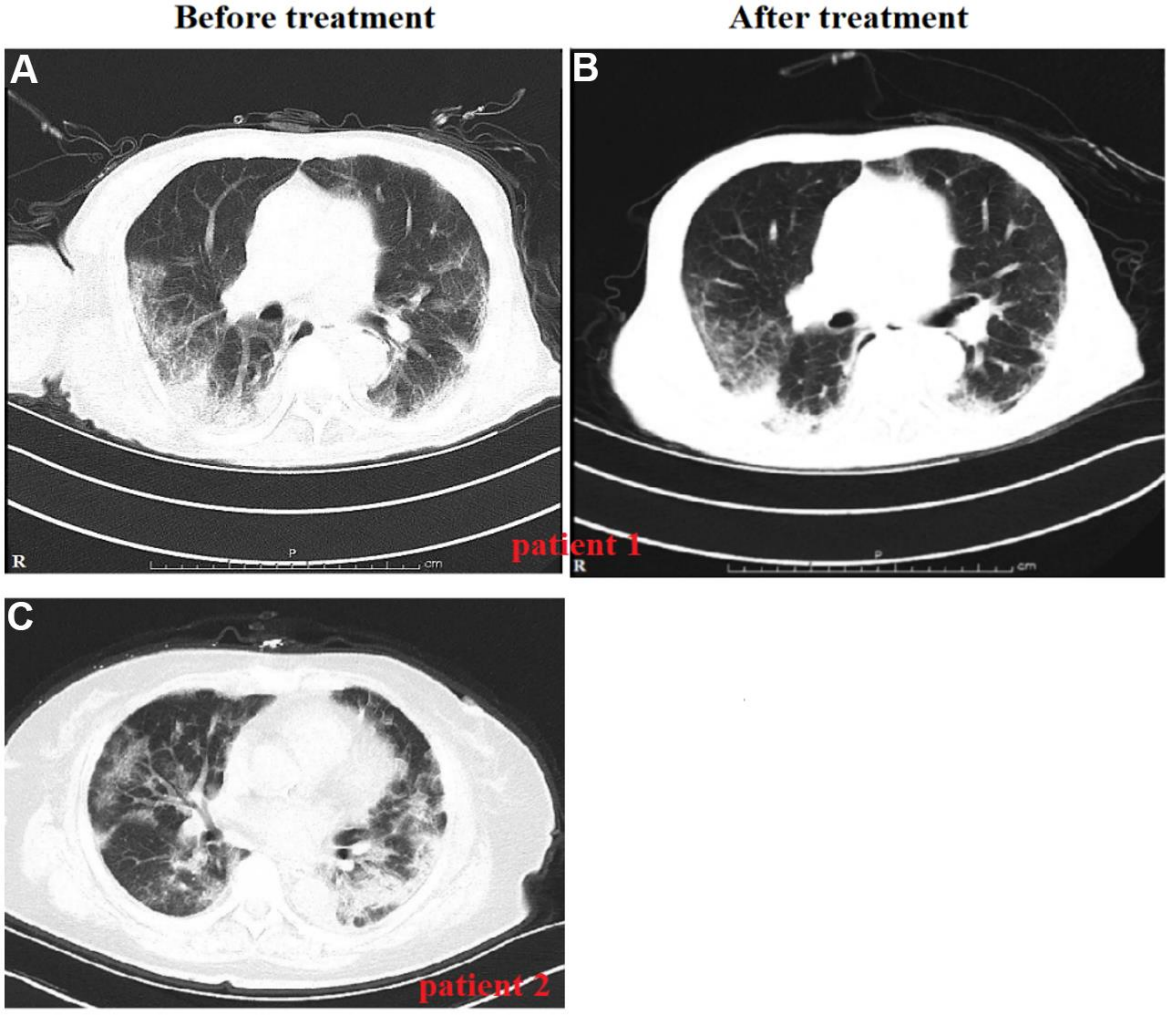
**Variation in radiological findings in patients with severe and critical COVID-19 during disease course.** Chest computed tomography (CT) shows severe multiple patchy shadows in both lungs on day 20 of symptom onset (examined upon admission) before treatment (**A**). Chest CT shows improved patchy shadows on day 39 of symptom onset for the same patient after treatment (**B**). Chest CT for a 68-year-old female who presented with high fever and dyspnea before treatment (**C**). Followed-up CT assessment was not performed because the patient died of her severe illness.

Eighty patients died during hospitalization. Table 2 displayed the characteristics of both the passed-away 80 patients (53.7%) and the survived 69 (46.3%) with severe or critical COVID-19 pneumonia. The elder (67.71±10.74 vs. 59.94±14.74, *p* < 0.001) and male (58/80 vs. 36/69, *p* = 0.010) patients died. Moreover, the SaO_2_, systolic blood pressure, breath rate, heart rate, and most blood biochemical indicators (e.g., WBC, lymphocytes, platelets, blood urea nitrogen, creatinine, eGFR, ALT, AST, albumin, TBil, and LDH) were significantly different between the patients who died and those who survived. Computed tomography (CT) is essential for COVID-19; the three most common pulmonary features on chest CT were bilateral patchy shadowing, ground-glass opacity, and pleural thickening (Table 3). Chest CT showed unilateral or bilateral opacity lesions, which were also described as ground-glass opacity in the lungs of severe or critical cases, as well as the slow absorption of lesions, which are one of the primary causes for dyspnea in the patients (Figure 1).

**Table 2:** Clinical characteristics of patients with COVID-19.

**Characteristics**	**Survival N=69**	**Death N=80**	***P* value**
Age (years)	59.94±14.74	67.71±10.74	<0.001
Sex(M/F)	36/33	58/22	0.010
DM (%)	20.3	13.9	0.302
Hypertension (%)	34.8	45.6	0.182
COPD (%)	7.2	6.3	0.824
CAD (%)	11.6	17.7	0.296
SaO_2_ (%)	94.62±4.20	84.81±13.88	<0.001
Fever (%)	85.5	91.3	0.272
Cough (%)	62.3	73.8	0.134
Chest distress/pain (%)	18.8	32.5	0.059
Dyspnea (%)	24.6	43.8	0.015
Fatigue (%)	21.7	37.5	0.037
Dizzy/headache (%)	7.2	7.5	0.953
Nausea (%)	1.4	3.8	0.386
Breathe(times/min)	22.33±4.99	26.85±7.06	<0.001
Heart rate(times/min)	92.38±16.88	99.71±19.99	0.018
Systolic blood pressure (mmHg)	128.75±18.35	135.39±21.35	0.046
Diastolic blood pressure(mmHg)	80.39±13.75	79.60±12.85	0.717
WBC (×10^9^/L)	7.69±4.12	11.10±6.59	<0.001
Neutrophil (×10^9^/L)	5.96±3.54	9.98±6.23	<0.001
Lymphocyte (×10^9^/L)	0.99±0.49	0.57±0.34	<0.001
Hemoglobin (g/L)	123.13±19.81	126.91±22.79	0.285
Platelet (×10^9^/L)	224.39±98.99	169.84±87.81	<0.001
Blood urea nitrogen(mmol/L)	6.53±6.00	12.23±10.63	<0.001
Creatinine(μmol/L)	71.94±29.63	116.26±104.48	<0.001
eGFR (ml/min/1.73m^2^)	87.84±26.56	69.69±29.37	<0.001
Blood uric acid (μmol/L)	244.35±99.90	292.38±191.92	0.053
Alanine aminotransferase(U/L)	24(6-221)	30.5(7-705)	0.032^a^
Aspartate aminotransferase (U/L)	31(10-189)	44(10-2368)	<0.001^a^
Albumin (g/L)	34.13±4.93	29.88±4.63	<0.001
ALP(IU/L)	80.00±37.71	90.23±43.79	0.132
TBil (μmol/L)	9.4(3.3-49.0)	12.6(3.2-174.1)	0.002^a^
Total cholesterol (mmol/L)	3.71±0.98	3.46±0.98	0.118
LDH (IU/L)	343.45±166.78	613.34±351.58	<0.001
TNF-α(pg/ml)	8.1(4.0-22.7)	10.35(4.0-69.7)	<0.001 ^a^
IL-6(pg/ml)	14.3(1.5-5000)	59.8(2.8-5000)	<0.001 ^a^
IL-8(pg/ml)	12.7(5.0-462.0)	26.6(6.4-1045)	<0.001 ^a^
IL-1β(pg/ml)	5.0(5.0-82.0)	5.0(5.0-7.2)	0.027 ^a^
IL-10(pg/ml)	5.0(5.0-61.0)	11.4(5.0-73.7)	<0.001 ^a^
Endotracheal intubation (%)	4(5.8)	9(11.2)	<0.001

^a^: Used Mann-Whitney U test.

Abbreviations: SaO_2_, oxygen saturation; eGFR, estimated glomerular filtration rate; COPD, chronic obstructive pulmonary disease; CAD, coronary artery disease; DM, diabetes mellitus; WBC, white blood cells; ALP, alkaline phosphatase; TBil, total bilirubin; LDH, lactate dehydrogenase; CI, confidence interval.

**Table 3:** Computed tomography features of patients with COVID-19.

**Chest CT findings**	**Total N=83**	**Severe N=40**	**Critical N=43**	**Survival N=60**	**Death N=23**
Ground glass opacity	41	20	21	29	12
Local patchy shadowing	1	0	1	1	0
Bilateral patchy shadowing	69	38	31	53	16
Mediastinal lymph nodes enlargement	13	9	4	13	0
Pleural effusion	11	0	11	6	5
Pleural thickening	29	18	11	26	3
Local bronchiectasis	4	2	2	4	0
Local pulmonary consolidation	11	6	5	10	1
White lung	2	0	2	1	1

### Cytokine test results

**Figure 2:**
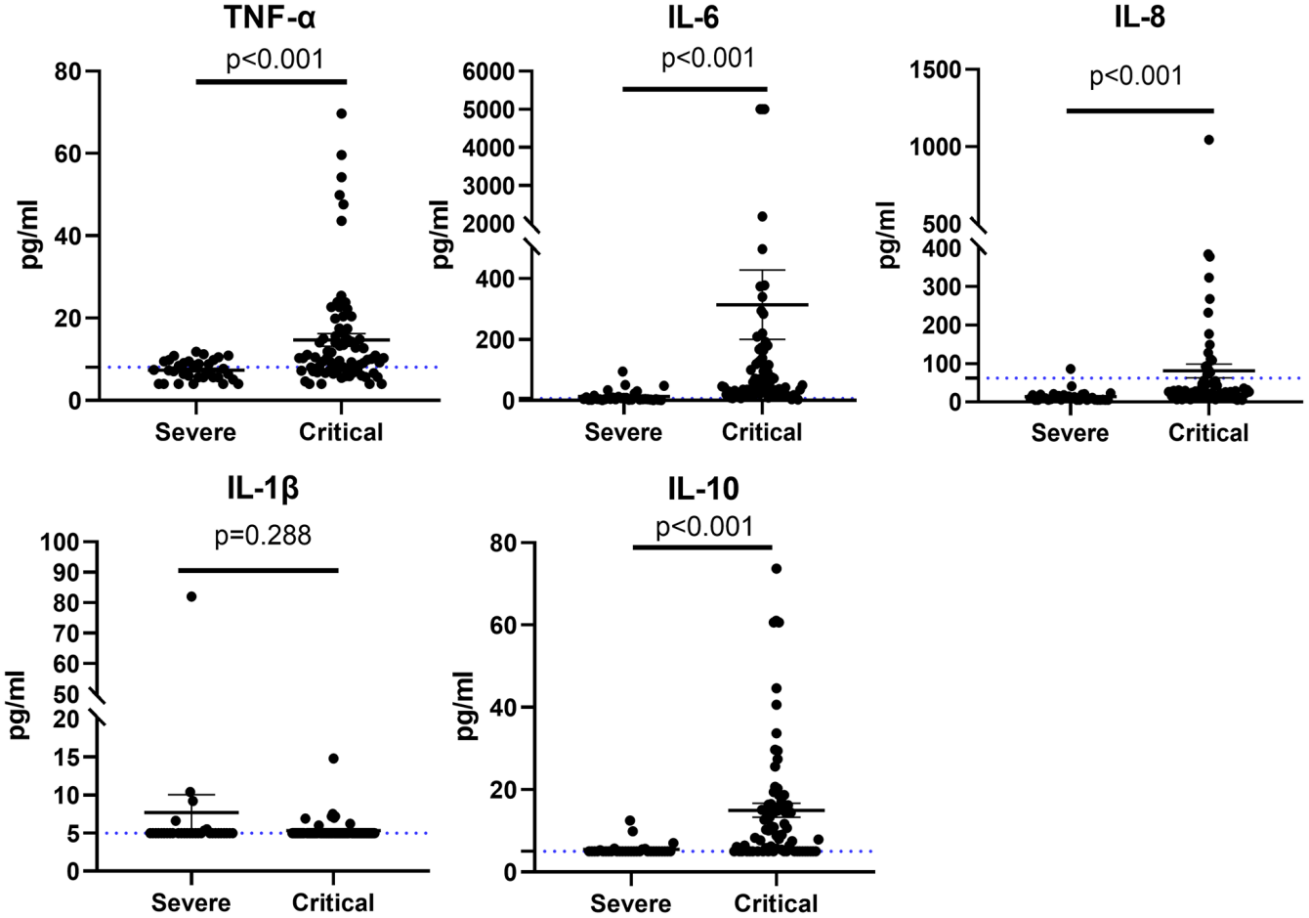
Cytokine profile of patients with severe or critical COVID-19 for TNF-α, IL-6, IL-8, IL-1β, and IL-10.

**Figure 3:**
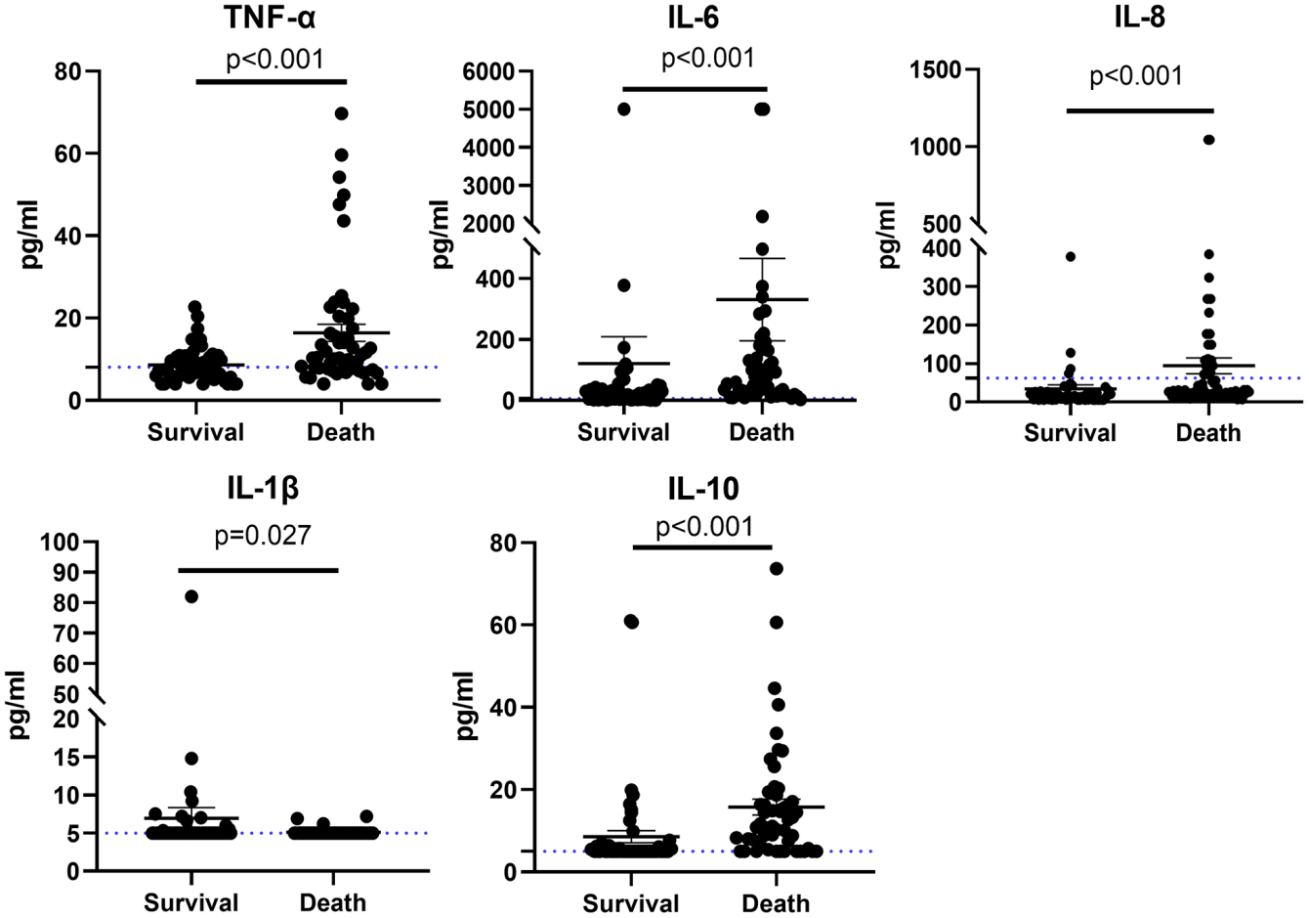
Cytokine profile of patients with COVID-19 who survived or died for TNF-α, IL-6, IL-8, IL-1β, and IL-10.

Serum cytokine results of 107 patients were exhibited in Figure 2. Of these proinflammatory cytokines (IL-1β, IL-6, IL-8, and TNF-α), the baseline levels of these cytokines were within normal range in most severe patients, whereas the levels of IL-8, TNF-α, and IL-6 significantly increased in most critical patients. IL-10 is a classic anti-inflammatory cytokine; the level of IL-10 was higher in the critical cases. The baseline levels of serum cytokine results between the two groups were also evaluated; the levels of IL-8, TNF-α, IL-6, and IL-10 elevated in patients who died, and whereas the level of IL-1β in the patients who died was lower than that of those who survived (Figure 3).

The results of Cox regression analyses for all-cause death are summarized in Table 4. After adjusting for age and sex, serum TNF-α level turned out to be associated with all-cause death (1.039 [1.019–1.059]; *p*<0.001). After similar adjustment for potential explanatory variables (i.e., age, sex, breath rate, heart rate, SaO_2_, systolic blood pressure, alanine aminotransferase, eGFR, COPD, CAD, and DM), serum TNF-α acted as an independent risk factor in this model for the mortality among patients with COVID-19 (1.047 [1.014–1.082]; *p* = 0.006). The relationship between the other proinflammatory cytokines and all-cause death was also assessed. Among all classic cytokines, only TNF-α was considered to be an independent risk factor for the death of the enrolled patients (Supplementary Tables 1–4).

**Table 4:** Cox regression analysis of death among patients with severe and critical COVID-19.

**Variable**	**Hazards Ratio**	**95%CI**	***P* value**
Model 1			
Sex(M/F)	1.047	0.544-2.016	0.890
Age(years)	1.047	0.020-1.074	0.001
TNF-α(pg/ml)	1.039	1.019-1.059	<0.001
Model 2			
Sex(M/F)	1.099	0.520-2.321	0.805
Age(years)	1.028	0.988-1.070	0.177
Breathe(times/min)	0.999	0.952-1.049	0.979
Heart rate(times/min)	0.991	0.971-1.012	0.405
SaO_2_(%)	0.952	0.925-0.981	0.001
Systolic blood pressure (mmHg)	1.005	0.989-1.021	0.565
Alanine aminotransferase(U/L)	1.001	0.998-1.004	0.627
eGFR(ml/min/1.73m^2^)	0.994	0.975-1.014	0.558
COPD	3.700	0.960-14.250	0.057
CAD	1.188	0.492-2.860	0.702
DM	1.608	0.694-3.725	0.268
TNF-α (pg/ml)	1.047	1.014-1.082	0.006

Abbreviations: SaO_2_, oxygen saturation; eGFR, estimated glomerular filtration rate; COPD, chronic obstructive pulmonary disease; CAD, coronary artery disease; DM, diabetes mellitus; TNF-α, tumor necrosis factor α; CI, confidence interval.

Model 1: adjusted for age and sex.

Model 2: adjusted for age, sex, breathe, heart rate, SaO2, systolic blood pressure, alanine aminotransferase, eGFR, COPD, CAD, and DM.

In terms of severity of conditions, age, sex and/or the potential variables were adjusted and later analyzed via logistic regression analyses (Table 5). TNF-α level was also turned out to be associated with the severity of COVID-19 patients (1.300 [1.086–1.556]; *p* = 0.004; 1.476 [1.136–1.919]; *p* = 0.004).

**Table 5:** Logistic regression analysis of illness severity among patients with COVID-19.

**Variable**	**Odds ratio**	**95%CI**	***P* value**
Model 1			
Sex(M/F)	0.607	0.199-1.847	0.379
Age(years)	1.125	1.063-1.191	<0.001
TNF-α(pg/ml)	1.300	1.086-1.556	0.004
Model 2			
Sex(M/F)	1.045	0.253-4.320	0.951
Age(years)	1.127	1.039-1.223	0.004
Breathe(times/min)	1.044	0.882-1.237	0.615
Heart rate(times/min)	1.013	0.959-1.070	0.639
SaO_2_(%)	0.848	0.756-0.952	0.005
Systolic blood pressure (mmHg)	1.029	0.992-1.067	0.128
Alanine aminotransferase(U/L)	1.017	0.990-1.044	0.214
eGFR(ml/min/1.73m^2^)	0.982	0.946-1.019	0.328
COPD	3.372	0.191-59.582	0.407
CAD	3.399	0.377-30.624	0.275
DM	0.701	0.120-4.093	0.693
TNF-α(pg/ml)	1.476	1.136-1.919	0.004

Abbreviations: SaO2, oxygen saturation; eGFR, estimated glomerular filtration rate; COPD, chronic obstructive pulmonary disease; CAD, coronary artery disease; DM, diabetes mellitus; TNF-α, tumor necrosis factor α; CI, confidence interval.

Model 1: adjusted for age and sex.

Model 2: adjusted for age, sex, breathe, heart rate, SaO2, systolic blood pressure, alanine aminotransferase, eGFR, COPD, CAD, and DM.

## DISCUSSION

In our present study, we explored the correlation between increasing TNF-α level and in-hospital death in patients with severe or critical COVID-19 pneumonia. We found that level of TNF-α was higher in critical patients than that in severe cases. And independent of other proinflammatory cytokines, TNF-α was a risk factor for the mortality of COVID-19 infection based on logistic regression analyses, indicating that TNF-α could be a potential treatment target for severe or critical COVID-19 pneumonia cases.

The decline number of peripheral lymphocytes is a typical feature of COVID-19 infection, an attribute that is also included in the diagnostic criteria of COVID-19 in China. After SRAS-CoV-2 infected, severe lymphopenia is a very early sign ahead of pulmonary lesions, and it will normalize along with the improvement of the disease [11]. The results of this study also supported this observation. In this study, the mean lymphocyte was 0.77±0.47×10^9^/L, and the value was lower in critical patients than in severe cases. In partially, the abnormal T lymphocyte count reflects the degree of immunologic dissonance after SARS-CoV-19 infection. A previous study showed that the decline of CD8+ T cell count is greater than count of CD4+ T cell, and the decline in the former is positively related with poor prognosis of patients with COVID-19 [12]. Normally, the CD8+ cytolytic T cells could secrete the granulysin to dissolve the virus-infected cells, resulting in the apoptosis of antigen presenting cells to avoid excessive activation after the antigen recognition is over [4]. In consequence, the lack of lymphocyte cytolytic activity is likely to exaggerate the activity of immune cells, and resulting in produce an excess of proinflammatory cytokines which thereby leading to cytokine storm.

In this study, levels of serum proinflammatory cytokines increased in most patients with COVID-19, and the level of most cytokines (e.g., TNF-α, IL-6, and IL-8) were greatly higher in critical patients than in severe cases. This result indicated that the levels of these proinflammatory cytokines were associated with disease severity. Autopsy results demonstrated that the quantity of infiltrated monocytes and macrophages in damaged lung are associated with the alveolar injury [7]. Therefore, monocyte- or macrophage-related cytokines (e.g., IL-1β, TNF-α, IL-6, and IL-8) dominate cytokine storm or target organ injury. It is vital for the design of future therapies to understand the mechanism of the immune response in order to reduce cytokine storm. Targeted immune cell-based therapies proved benefits among patients as they are targeted at a specific cytokine without causing a widespread effect on the immune system [13].

Indeed, this study showed profoundly higher levels of TNF-α, IL-6, IL-8, and IL-10 in critical cases or patients who died, which is consistent with the findings of previous studies [10, 14, 15]. IL-6 is a pleiotropic cytokine. It is crucial to regulate immunological and inflammatory responses [16]. Previous studies showed that the elevation of serum IL-6 was considered to be related with the severity of COVID-19 [14, 17]. In this study, the serum IL-6 level went up among critical patients, but further analyses did not show that IL-6 is an independent risk factor. Based on guideline for diagnosis and management of COVID-19, the participants in this study were all suffered from severe or critical conditions [9]. Therefore, we believe that the different conditions among participant and evaluative criteria is highly likely to be the reason why the results in this study were partially different from the previous findings [14, 17]. Cauchois R and his colleagues demonstrated that SARS-CoV-2 could induce a secretion of active IL-1β and IL-18 to initiate the cytokine release syndrome through the activation of NLRP3 inflammasome, and early blockade of the IL-1β receptor with anakinra could arrest the deterioration of the patient’s condition and reduce the need for invasive mechanical ventilation [18]. However, we did not find a significant increase in the level of serum IL-1β in severe or critical COVID-19 patients. Because of the characteristic describe above, IL-1β is difficulty to isolate from peripheral blood. Moreover, most patients in severe or critical condition in ICU have to go through a long diagnosis or examination time. Thus, the majority of patients in this study had low IL-1β levels below the detection threshold (<5 pg/mL). Certainly, it is undoubtful that IL-1β plays a key role in the pathological process of COVID-19. Previous studies showed that IL-1β is able to induce IL-6 immune response in inflammatory diseases, and the decline of IL-1β can consistently reduce the circulating levels of IL-6 [19, 20]. Therefore, it can be concluded that rapid activation of IL-1β plays an important role in the initialization phase of COVID-19 infection.

The incidence of acute respiratory distress syndrome in patients with COVID-19 is from 14.8% to 17% [21, 22], and 61.1% in patients in ICU [23]. High levels of proinflammatory cytokines (e.g., IL-12, IL-7, monocyte chemoattractant protein-1 [MCP-1], TNF-α, and granulocyte colony-stimulating factor) are essential in the pathogenesis of COVID-19 [10]. Patients with severe disease exhibit higher serum levels of IL-2R, IL-6, and TNF-α than those with a mild or moderate disease [24, 25]. Although cytokine storm and the release of inflammatory factors have been studied in different diseases, the downstream signals and the pathway of subsequent inflammatory diffusion remain unclear. Cytokine storm is not uncommon existent in COVID-19 pathology and is considered one of the hallmarks. The cytokine storm induced by SRAR-CoV-2 is the pathological feature of most severe cases. A recent study reported that inhibition of TNF-α and IFN-γ could improve death rate caused by COVID-19 infection, sepsis, and cytokine shock [26]. Of the cytokines analyzed herein, the proinflammatory cytokine and its relationship with COVID-19 mortality have been assessed. After potential explanatory variables were adjusted, the TNF-α was the only independent risk factor for death and the severity of disease. Rajendra [26] supports our findings and partially share the same explanation about the molecular mechanism that TNF-α is an independent hazardous factor for COVID-19 related death and disease severity.

The activation of NF-κB and IRF3 transcriptional activity have been identified in the downstream signal pathway after SARS-CoV being recognized by specific receptor [27]. This early-stage process results in the synthesis of type I IFN and proinflammatory cytokines, and then prevents against the virus invasion. SARS-CoV-2 induces an immune reaction through infiltration of many immune cells, leading to a high production of cell factors including IL-6, IL-8, IL-10, TNF-α, MCP-1, CRP, and ferritin, and ultimately forms the cytokine storm [28]. Unsurprisingly, cytokine storm described above shares the similar feature with SARS-CoV and MERS-CoV pneumonia [29, 30]. A study indicated that elevation of IL-6 could be a biomarker for severity assessment or a prognostic indicator in patients with pneumonia, the similar tendency can be discovered in the balance between IL-6 and IL-10 [31]. During the H5N1 avian influenza period, a sharply increase of circulating cytokines (e.g., IL-6, IL-2 and IFN-γ) could be a potential explanation for lymphocyte depletion in died patients. Furthermore, excessive activation of immune system leads to the hypercytokinemia, which can contribute to influenza pathology in humans, chickens, and mice [32]. The impaired cytokine and/or chemokine responses are partially responsible for the severity of human H5N1 infection [33]. The results of cytometric bead array indicated that concentrations of IP-10, MIC, IL-6, MCP-1, IL-8, and IFN-α were markedly higher in patients with avian influenza A than those in health controls [33]. This result suggested that cytokines are closely related to the development of pulmonary virus infection. Plenty of cytokines and/or chemokines release continuously as long as the excessive activation of the immune response, which can in turn break down lung tissues, deteriorate respiratory capacity, and result in severe disease and death.

TNF-α is a strong proinflammatory cytokine that plays a key role during inflammation, cell proliferation, differentiation, and apoptosis [34]. TNF-α is secreted mainly by macrophages and T-cells as it is used depending on circumstance. However, stimulations will activate its de novo synthesis. TNF-α-induced cell death depends on its receptor, namely, –TNFR1, containing a specific death domain. In resting state, the death domain signaling pathway fails to trigger due to the association of receptor and a cytoplasmic silencer of the death domain without TNF-α participation. While the silencer of the death domain will be separated away from receptor as soon as TNF-α activated and then they can interact with the other death domain on another adaptor protein [35, 36].

During COVID-19 infection, TNF-α-derived inflammatory cascade is responsible for lung damage, and the latter can be markedly reduced by inhibiting TNF-α. Not only TNF-α inhibition can improve the virus-specific lung injury in mice, but also anti-TNF antibody relieve the overall severe illness condition independent of viral clearance [37]. In humans, as an anti-TNF antibody, etanercept is used to treat noninfectious idiopathic pneumonia syndrome which similar to virus-related pneumonia in some ways [38].

In addition to vaccine prevention, high-efficiency strategies for fighting COVID-19 are still be needed. In view of the urgency to address the current health crisis, remedial treatment must be rapidly developed. Therefore, rediscovery and drug repurposing of existing drugs could help improve disease development in clinical trials [39, 40]. The safety profile of these medicines confers them an advantage over newly designed therapy. The severity of SARS may relate to increasing age by showing that the older generally presented higher TNF-α secretion than younger participants [41]. Blockade of TNF-α can dramatically improve disease severity and lung damage by interrupting virus-derived inflammatory storm without affecting viral clearance. Among the three approved anti-TNF biologics including infliximab, adalimumab, and etanercept, the latter would be preferentially selected in deference to its safety, short half-life, and especially low efficacy immunogenicity. However, inhibition of innate antiviral defense may be the potential side effect of TNF-α inhibitors. Real evidence supporting the presumption that TNF-α inhibitor makes patients susceptible to being infected is lacking, but clinical experience from the use of anti-TNF-α in virus-specific lung immunopathology supports the effects of the TNF-α inhibitors described above [37]. The lung injury of patients with severe COVID-19 is at least partially mediated by the immune response against the virus, and the regulation of inflammation might even play a protective role [42]. A case report on using the anti-TNF-α antibody for treating COVID-19 pneumonia and severe ulcerative colitis suggested that anti-TNF-α agents are an effective and safe therapy for COVID-19 [43].

The use of TNF blocker is clinically effective in many diseases, especially for several autoimmune cases, despite the existence of other proinflammatory cytokines and intermediary factors [44]. Therefore, the effectiveness of TNF inhibitors in patients with moderate COVID-19 should be initially assessed as soon as possible upon hospital admission [44]. A randomized controlled trail to evaluate adalimumab, an anti-TNF agent, for COVID-19 treatment has been registered (ChiCRT2000030089).

The study has several limitations. First, the enrolled patients were from a single center with limited samples. Second, other inflammatory cytokines (e.g., IFN, IL-17, IL-12, IL-18, and IL-4) and their relationship with mortality were not evaluated because of the lack of relevant data. Furthermore, the cytokine changes throughout the hospitalization duration were not analyzed because most of the patients underwent a single cytokine test only. Third, safety evaluation in large clinical studies was lacking while which should be evaluated in our further studies on COVID-19. Fourth, due to the continuous improvement of treatment standards at the beginning of the outbreak and lack of collecting data regarding treatments, we fail to observe the different treatment strategies among the enrolled patients. Finally, due to the emphasis of our study were severe or critical cases only, the potential role of TNF-α in disease prognosis might not be directly applicable to non-severe cases. However, cytokine storm tends to occur in severe cases. Thus, mild or moderate cases often do not need immunosuppression treatment. Therefore, exploring the TNF-α level in patient with mild or moderate COVID-19 pneumonia may be less valuable.

The characteristics of patients with COVID-19 in severe or critical condition were estimated by performing a common serum cytokine test. Serum TNF-α level was found to be an independent risk factor for the mortality of patients with severe or critical COVID-19. We concluded that serum TNF-α level acts as an independent risk factor for the mortality of severe and/or critical COVID-19 patients. Therefore, anti-TNF-α treatment for severe COVID-19 patients could be a better choice to improve the mortality rate.

## Supplementary Material

Supplementary Tables
