# Supplementary material for: Role of tumor necrosis factor-α in the mortality of hospitalized patients with severe and critical COVID-19 pneumonia
Source: Aging (Albany NY). 2021 Nov 1;13(21):23895–912. doi: 10.18632/aging.203663 (PMC8610114; doi:10.18632/aging.203663)
Supplement: Supplementary Tables [file aging-13-203663-s001.pdf]

SUPPLEMENTARY TABLES

Supplementary Table 1. Cox regression analysis of death among patients with severe and critical COVID-19.

Variable	Hazard ratio	95%CI	*P* value
Model1			
Sex(M/F)	1.104	0.572-2.130	0.768
Age(years)	1.046	1.020-1.072	<0.001
IL-1β (pg/ml)	0.527	0.214-1.295	0.163
Model2			
Sex(M/F)	1.115	0.513-2.427	0.783
Age(years)	1.014	0.979-1.051	0.426
Breathe(times/min)	1.006	0.952-1.063	0.821
Heart rate(times/min)	1.002	0.983-1.021	0.850
SaO_2(%)	0.960	0.931-0.990	0.008
Systolic blood pressure (mmHg)	1.007	0.989-1.026	0.463
Alanine aminotransferase(U/L)	0.999	0.996-1.002	0.554
eGFR(ml/min/1.73m^2)	0.981	0.967-0.994	0.005
COPD	2.169	0.583-8.076	0.248
CAD	1.112	0.474-2.610	0.807
DM	2.070	0.905-4.735	0.085
IL-1β(pg/ml)	0.487	0.183-1.297	0.150

Model 1: different cytokine adjusted for age and sex.
Model 2: different cytokine adjusted for age, sex, breathe, heart rate, SaO2, systolic blood pressure, alanine aminotransferase, eGFR, COPD, CAD, and DM.
Abbreviations: SaO2, oxygen saturation; eGFR, estimated glomerular filtration rate; COPD, chronic obstructive pulmonary disease; CAD, coronary artery disease; DM, diabetes mellitus; CI, confidence interval, IL interleukin.

Supplementary Table 2. Cox regression analysis of death among patients with severe and critical COVID-19.

Variable	Hazard ratio	95%CI	*P* value
Model1			
Sex(M/F)	0.986	0.518-1.879	0.967
Age(years)	1.047	1.020-1.075	0.001
IL-6 (pg/ml)	1.000	1.000-1.000	0.793
Model2			
Sex(M/F)	1.041	0.496-2.187	0.915
Age(years)	1.013	0.977-1.051	0.477
Breathe(times/min)	0.986	0.941-1.033	0.558
Heart rate(times/min)	0.999	0.980-1.018	0.884
SaO_2(%)	0.952	0.922-0.982	0.002
Systolic blood pressure (mmHg)	1.004	0.987-1.021	0.644
Alanine aminotransferase(U/L)	1.000	0.996-1.003	0.835
eGFR(ml/min/1.73m^2)	0.979	0.966-0.992	0.002
COPD	2.547	0.685-9.471	0.163
CAD	1.059	0.432-2.596	0.900
DM	2.458	1.089-5.548	0.030
IL-6 (pg/ml)	1.000	1.000-1.000	0.325

Model 1: different cytokine adjusted for age and sex.
Model 2: different cytokine adjusted for age, sex, breathe, heart rate, SaO2, systolic blood pressure, alanine aminotransferase, eGFR, COPD, CAD, and DM.
Abbreviations: SaO2, oxygen saturation; eGFR, estimated glomerular filtration rate; COPD, chronic obstructive pulmonary disease; CAD, coronary artery disease; DM, diabetes mellitus; CI, confidence interval, IL interleukin.

Supplementary Table 3. Cox regression analysis of death among patients with severe and critical COVID-19.

Variable	Hazard ratio	95%CI	*P* value
Model1			
Sex(M/F)	0.972	0.497-1.899	0.933
Age(years)	1.046	1.020-1.073	<0.001
IL-8 (pg/ml)	1.001	0.999-1.002	0.258
Model2			
Sex(M/F)	0.922	0.418-2.033	0.840
Age(years)	1.017	0.981-1.055	0.354
Breathe(times/min)	0.989	0.943-1.036	0.632
Heart rate(times/min)	1.000	0.981-1.019	0.981
SaO_2(%)	0.954	0.926-0.982	0.002
Systolic blood pressure (mmHg)	1.003	0.985-1.020	0.778
Alanine aminotransferase(U/L)	0.999	0.996-1.003	0.719
eGFR(ml/min/1.73m^2)	0.979	0.966-0.993	0.003
COPD	2.473	0.677-9.029	0.171
CAD	1.101	0.462-2.622	0.828
DM	2.486	1.091-5.669	0.030
IL-8(pg/ml)	1.001	0.999-1.003	0.211

Model 1: different cytokine adjusted for age and sex.
Model 2: different cytokine adjusted for age, sex, breathe, heart rate, SaO2, systolic blood pressure, alanine aminotransferase, eGFR, COPD, CAD, and DM.
Abbreviations: SaO2, oxygen saturation; eGFR, estimated glomerular filtration rate; COPD, chronic obstructive pulmonary disease; CAD, coronary artery disease; DM, diabetes mellitus; CI, confidence interval, IL interleukin.

Supplementary Table 4. Cox regression analysis of death among patients with severe and critical COVID-19.

Variable	Hazard ratio	95%CI	*P* value
Model1			
Sex(M/F)	1.017	0.526-1.968	0.960
Age(years)	1.042	1.015-1.070	0.002
IL-10 (pg/ml)	1.013	0.995-1.032	0.161
Model2			
Sex(M/F)	0.946	0.438-2.041	0.887
Age(years)	1.010	0.974-1.049	0.584
Breathe(times/min)	0.985	0.940-1.032	0.529
Heart rate(times/min)	0.998	0.980-1.018	0.875
SaO_2(%)	0.951	0.923-0.980	0.001
Systolic blood pressure (mmHg)	1.004	0.986-1.022	0.681
Alanine aminotransferase(U/L)	0.999	0.996-1.002	0.475
eGFR(ml/min/1.73m^2)	0.980	0.966-0.994	0.004
COPD	2.988	0.785-11.369	0.108
CAD	1.060	0.444-2.529	0.896
DM	2.548	1.118-5.807	0.026
IL-10(pg/ml)	1.022	0.999-1.044	0.059

Model 1: different cytokine adjusted for age and sex.
Model 2: different cytokine adjusted for age, sex, breathe, heart rate, SaO2, systolic blood pressure, alanine aminotransferase, eGFR, COPD, CAD, and DM.
Abbreviations: SaO2, oxygen saturation; eGFR, estimated glomerular filtration rate; COPD, chronic obstructive pulmonary disease; CAD, coronary artery disease; DM, diabetes mellitus; CI, confidence interval, IL interleukin.
